# BMPER induces the adipogenic differentiation of fibro/adipogenic progenitors and promotes intramuscular fat deposition in chickens

**DOI:** 10.1186/s40104-026-01389-9

**Published:** 2026-04-19

**Authors:** Ying Liu, Jianliang Liu, Xuan Sun, Xiuze Zhang, Tong Xing, Lin Zhang, Feng Gao, Liang Zhao

**Affiliations:** 1 College of Animal Science and Technology, State Key Laboratory of Meat Quality Control and Cultured Meat Development, Key Laboratory of Animal Origin Food Production and Safety Guarantee of Jiangsu Province, Jiangsu Collaborative Innovation Center of Meat Production and Processing, Quality and Safety Control, Nanjing Agricultural University, No. 1 Weigang, Nanjing, 210095 P.R. China; 2 College of Food Science and Technology, Nanjing Agricultural University, Nanjing, 210095 P.R. China

**Keywords:** Adipogenic progression, BMPER, Broilers, Fibro/adipogenic progenitors, snRNA-seq

## Abstract

**Background:**

Fibro-adipogenic progenitors (FAPs) serve as the developmental origin of intramuscular adipocytes in skeletal muscle‌. Enhancing their adipogenic transition could increase intramuscular fat (IMF) deposition, thereby improving meat flavor in chickens‌. However, critical aspects of FAPs including their cellular composition, dynamic changes during skeletal muscle growth, and associated regulatory mechanisms remain poorly understood‌. Therefore, we comprehensively characterized FAPs in Langshan chickens from d 1 to d 98 using both in vivo and in vitro approaches combined with single-nucleus RNA sequencing (snRNA-seq) analysis.

**Results:**

Our analysis of IMF contents and adipogenesis-related gene expression in the pectoralis major muscle of Langshan chickens revealed that the adipogenic properties of FAPs peaked at d 1, reached its lowest point at d 14, and subsequently increased until d 98. The snRNA-seq analysis successfully identified the population of FAPs along with their 5 subtypes including the pre-adipogenic, adipogenic, and fibrotic FAPs. The ratio of the pre-adipogenic subtype decreased from d 14 to d 98, which was reversely correlated with the changes of the adipogenic subtype, suggesting a differentiating process. Furthermore, RNA velocity and pseudo-trajectory analysis revealed that the initial FAPs had superior fibrotic capacity but decreased over time which contrasted with their enhanced adipogenic capacity with development. Notably, BMPER was identified as an important regulator for the adipogenic differentiation of FAPs, which was also confirmed by in vitro over-expression studies. In addition, the expression of *BMPER* in the adipogenic portion of FAPs was found to be highly conserved across human and mouse skeletal muscles.

**Conclusions:**

Our study provides the first comprehensive atlas of FAPs in the skeletal muscle of chickens and identifies BMPER as a key regulator for the adipogenic differentiation of FAPs. The findings will not only provide novel targets for breeding chickens with high IMF content but also offer significant insights into understanding the cell fate decision of FAPs under both physiological and pathological conditions across species.

**Supplementary Information:**

The online version contains supplementary material available at 10.1186/s40104-026-01389-9.

## Introduction

Intensive genetic selection of broilers for growth rate and meat production has significantly increased production efficiency, resulting in a higher proportion of breast and leg muscles. Advances in animal nutrition and management have further supported this accelerated growth in meat yield. However, such rapid growth of broilers often results in decreased intramuscular fat (IMF) content, a key determinant of meat quality as increased IMF improves flavor and tenderness [1]. Therefore, to address this quality challenge and meet the market expectation for superior meat quality, producing local breeds with greater IMF deposition and enhanced flavor has become an important goal for the poultry industry. This direction is supported by research confirming that local breeds can offer distinct flavor advantages, and that meat quality traits like IMF are influenced by breed and production factors [2, 3].

The content of IMF in the skeletal muscle is determined primarily by the number of intramuscular adipocytes and their lipid deposition capacity [4, 5]. A group of multi-potential progenitors, known as fibro/adipogenic progenitors (FAPs), has been shown to be the developmental origin of intramuscular adipocytes [6, 7]. These FAPs express cell surface markers of PDGFRα and SCA1, and have the potential to differentiate into adipocytes, fibroblasts, and osteoblasts [6, 7]. Therefore, the biological process of IMF deposition requires the early adipogenic transition of FAPs into preadipocytes, followed by the maturation of preadipocytes and fat deposition in the form of lipid droplets [8–10]. Consequently, the abundance of FAPs in skeletal muscle and their intrinsic adipogenic abilities directly influence the final number of intramuscular adipocytes and IMF content. The proliferative and differentiation abilities of FAPs exhibit stage-specific characteristics during skeletal muscle growth [11]. In our previous work on beef cattle, we proposed that the period from birth to weaning (approximately 0–250 days of age) is critical for the proliferation of FAPs, whereas the fattening period is the phase for their adipogenic differentiation [11, 12]. Accordingly, injecting vitamin A at 0 or 1 month of age promoted the proliferation of FAPs and increased their density in the skeletal muscle of calf, while restricting vitamin A intake during the fattening period enhanced the adipogenic differentiation of FAPs [11, 13]. Through the above methods of nutritional regulations targeting FAPs, the marbling in beef cattle has been greatly enhanced [13, 14]. In broilers, a close relationship between *PDGFRA* gene expression and IMF deposition was also suggested [15, 16]. However, the dynamic changes in FAPs cellularity, including proliferation and adipogenic differentiation during skeletal muscle growth, and their underlying regulatory mechanisms remain barely understood in broilers.

While FAPs are established as the primary cellular source of IMF, their adipogenic differentiation competes with fibrotic fate determination. In studies of muscle regeneration, the TGFβ and PDGF signaling pathways were found to induce the fibrotic differentiation of FAPs, while the WNT5A/GSK-3/β-catenin, Notch2, and Interleukin 4 signaling pathways inhibited their adipogenic differentiation [17–21]. Previous studies from our laboratory have also demonstrated that retinoic acid signaling critically inhibits adipogenic differentiation of FAPs, while AMPKα1 signaling suppresses their fibrotic activation in the mouse skeletal muscle [22, 23]. In farm animals, promoting adipogenic while suppressing fibrotic differentiation of FAPs during muscle growth enhances intramuscular fat deposition and reduces connective tissue formation, ultimately improving meat quality. Unfortunately, there is a significant gap in research regarding the fate determination of FAPs in chicken muscles.

With advancements in high-throughput sequencing technology, researchers have leveraged single-cell/nucleus RNA sequencing (scRNA-seq or snRNA-seq) to comprehensively analyze the cellular composition of the longissimus dorsi muscle across cattle and pig breeds with varying IMF contents [24, 25]. This approach has successfully revealed the regulatory mechanisms of the proliferation and fate determination of FAPs across different breeds [24, 25]. However, comprehensive investigations about the dynamic cell fate determination and stage-specific characteristics of FAPs during muscle growth are still missing especially in chickens.

The meat-type Langshan chicken is a renowned Chinese breed whose superior meat quality comes with a longer production cycle, reaching commercial slaughter age at around 14 weeks. In this study, we first investigated the levels of lipid deposition, adipogenic gene expression, and population changes of TCF4^+^ FAPs across the growth period (d 1, d 14, d 28, d 42, d 70, and d 98) of Langshan chickens. Then, two representative ages from both the starter (d 1 and d 14) and grower stages (d 42 and d 98) were selected for snRNA-seq analysis in order to precisely characterize the dynamic changes of FAPs during muscle growth and elucidate their underlying regulatory mechanisms. In addition, our in vivo and in vitro studies identified BMPER as a critical regulator for the adipogenic differentiation of FAPs. Notably, this novel function of BMPER was likely conserved across species, as evidenced by our analysis of publicly accessible datasets of human and mouse muscle samples. Therefore, our findings will not only facilitate the breeding of broilers with high IMF content but also contribute to a broader understanding of muscle physiology across different species.

## Materials and methods

### Ethics statement

All experimental procedures and animal management of this study were performed according to the Guidelines for Experimental Animals established by the Institutional Animal Care and Use Committee of Nanjing Agricultural University (Approval No. NJAU.No20230516074).

### Experimental design and sample collection

A total of 200 one-day-old male Langshan chickens were reared under standardized conditions with ad libitum access to a commercial diet and water. Chickens were fed with a starter diet from d 1 to d 21, and then switched to a grower diet from d 22 until the end of the experiment at d 98 to meet their changing nutritional requirements (Table S1 in Additional file 1). At d 1, d 14, d 28, d 42, d 70, and d 98, eight chickens per time point were randomly selected (*n* = 8). Body weights were within the breed’s normal range with no inter-group differences. Birds were humanely euthanized to collect pectoralis major muscle samples. Briefly, chickens were placed in a CO_2_ chamber until loss of consciousness. Immediately thereafter, blood was collected from the jugular vein into 5-mL coagulation tubes, incubated at room temperature for 2 h, centrifuged at 3,000 × *g* for 10 min, and serum was stored at −80 °C. Cervical dislocation was subsequently performed to confirm death. Pectoralis major muscle was then rapidly excised and processed for single-nucleus transcriptomic analysis. From each chicken, the cranial portion of the right pectoralis major muscle (0.5 cm × 0.5 cm × 1 cm) was excised along the fiber direction to minimize compression and stretching damage and fixed in 4% paraformaldehyde for 24 h. The remaining pectoralis major muscle was flash-frozen in liquid nitrogen and stored at −80 °C.

### Measurement of IMF, triglyceride, and total cholesterol contents

To improve the accuracy of measurements, visible fat and fascia on the surface of the breast muscle samples were carefully removed before weighing. Each breast muscle sample was precisely weighed to 2.000 g (fresh weight). After freeze-drying, the IMF content was determined using the Soxhlet extraction method and expressed as a percentage of the dry weight of the breast muscle [26]. For triglyceride (TG) and total cholesterol (TC) measurements, each sample was accurately weighed (0.1 g) and homogenized with 0.9 mL of physiological saline. The clear supernatant from the homogenate was collected, and TG and TC levels were quantified using commercially available ELISA kits (Nanjing Jiancheng Bioengineering Institute, Nanjing, China).

### Immunofluorescence staining

The immunofluorescence staining of paraffin sections was conducted as previously described [23, 27]. The sections were first deparaffinized and rehydrated. Subsequently, antigen retrieval was performed using a microwave. The sections were then blocked with 3% BSA. For immunofluorescence, samples were incubated overnight at 4 °C with a rabbit polyclonal antibody against TCF4 (Proteintech, 22337-1-AP, 1:100). After washing, the sections were incubated for 50 min at room temperature with an HRP-conjugated goat anti-rabbit IgG antibody (Nanjing Dream Bio-Technology, YM0007-1, 1:100). The signal was developed using a fluorophore-conjugated tyramide reagent (Nanjing Dream Bio-Technology, YMTSA0002-520) according to the manufacturer's instructions. The cell nuclei were counterstained with DAPI for 10 min. When necessary, the autofluorescence of the tissue was quenched. Finally, the sections were mounted and observed under a fluorescence microscope.

### RNA extraction, cDNA synthesis, and RT-qPCR

Total RNA was extracted from broiler breast muscle tissues using RNAiso Plus reagent (Takara Biotechnology Co., Ltd., Dalian, China) and quantified using a NanoDrop ND-2000 spectrophotometer (Thermo Scientific, Pittsburgh, PA, USA). RNA (1 µg, with A_260_/A_280_ ratios of 1.8–2.0) was reverse transcribed into cDNA using the Uni All-in-One First-Strand cDNA Synthesis SuperMix (TransGen Biotech, Nanjing, China). qPCR was performed using PerfectStart Green qPCR SuperMix (TransGen Biotech) on an ABI QuantStudio 7 Flex system (Applied Biosystems, Foster City, CA, USA) in 20 µL reactions. The specific primer sequences for target and reference genes are listed in Table 1. The relative mRNA expression levels were calculated using the 2^-ΔΔCt^ method, with *ACTB* as the internal reference gene. All primers were synthesized by Tsingke Biotech (Nanjing, China).

**Table 1:** Primers used for RT-qPCR

Gene name	Accession number	Primer sequence (5′→ 3′)	Product size, bp
*BMPER*	XM_046911335.1	F: TCTCACTACCCTGCGAGACA R: TCATCTCGCTTGTGCCCATT	187
*CEBPA*	NM_001031459.2	F: CCTACGGCTACAGAGAGGCT R: TGATGTCGATGGAGTGCTCG	96
*CD36*	XM_040685898.2	F: AGGTACTGCGCTTCTTCTCC R: TCCAGCCAGTGTGCAGTT	194
*DLK1*	NM_001397238.1	F: TTTCTCCTTCTGTTCCGCCG R: AATCCATTCACTGGGTGGCA	280
*DGAT2*	XM_419374.8	F: GAACGAAGTGTACAAGCAGGTGA R: ATCGATCTTTGGGATGGTGATGG	202
*FABP4*	NM_001031459.2	F: GATGAGACCACAGCAGATGACAG R: TCCACCACTTTCCTCTTGATAACAG	119
*PDGFRA*	NM_204749.3	F: GCTTCCTCTGCCGACTATTGTTC R: CTGTTGTTCTCCTCGTGTCTGATG	159
*PPARG*	NM_001397666.1	F: CGAATGCCACAAGCGGAGAAG R: TGATTTGTCTGTCGTCTTTCCTGTC	186
*PCNA*	NM_204170.3	F: GACAATGCGGATACGTTGGC R: TCACCAATGTGGCTGAGGTC	188
*KLF7*	XM_046921657.1	F: ACTGCTTCCTTCATGCCTCCTC R: TCGCACATACTCGTCTCCACTG	94
*ZNF423*	XM_040681108.2	F:AGCCAGGAAGAGAGAAATGAGGAAG R: AGTCAGGTCTGCCAGCGAATC	102
*ACTB*	NM_205518	F: TCTTGGGTATGGAGTCCTG R: TAGAAGCATTTGCGGTGG	331

*BMPER* BMP-binding endothelial regulator, *CEBPA* CCAAT/enhancer-binding protein alpha, *CD36* Cluster of differentiation 36, *DLK1* Delta-like 1 homolog, *DGAT2* Diacylglycerol O-acyltransferase 2, *FABP4* Fatty acid-binding protein 4, *PDGFRA* Platelet-derived growth factor receptor alpha, *PPARG* Peroxisome proliferator-activated receptor gamma, *PCNA* Proliferating cell nuclear antigen, *KLF7* Krüppel-like factor 7, *ZNF423* Zinc finger protein 423, *ACTB* Actin, beta

### snRNA-seq library construction and sequencing

Nuclei were extracted from pooled samples of 200 mg pectoralis major muscle at each timepoint (3 broilers/time point). Specifically, nuclei were counted and assessed for viability using a Fluorescence Cell Analyzer (Countstar^®^ Rigel S2) with AO/PI staining. Morphological examination was performed under a 40 × microscope (Jiangnan Novel Optics XD-202) following staining with 0.4% trypan blue (Sangon Biotech, E607320-0001). Subsequent experimental steps were only carried out when nuclei displayed intact nuclear membranes and minimal impurities. After quality check, the snRNA-seq libraries were constructed following the standard protocol of SeekOne^®^ DD Single Cell 3′ library preparation kit (Catalog No. K00202) by Beijing SeekGene BioSciences [28]. Sequencing was performed on Illumina NovaSeq X Plus with PE150 read length.

### Processing of snRNA‑seq data

The raw sequencing data was processed by the Fastp package (v3.2.1) to trim primer sequences and low-quality bases. Subsequently, Seeksoul tools (Seekgene, v1.2.1) were used to process the resting data, demultiplex cellular barcodes and map reads to the chicken Gallus_gallus_GRCg7b reference genome. The generated feature-barcode matrices were imported into the R package of Seurat (v5.3.0) and analyzed according to the standard workflow as previously established in our laboratory [29, 30]. Matrices from the four groups were combined into one R object. For quality control, nuclei with nFeature_RNA < 500 genes and nCount_RNA > 25,000 UMIs were removed. After filtering, 66,662 nuclei were retained for downstream analysis. The resulting dataset was normalized using the LogNormalize method and scaled and reduced in dimensionality using the ScaleData and RunPCA functions. Next, the Harmony package was employed to integrate the expression matrices, a widely used method for mitigating batch effects introduced during sample preparation and sequencing. Cell clustering was performed using functions of RunUMAP, FindNeighbors, and FindClusters. Cell types were annotated based on marker genes identified by the function of FindAllMarkers. The core R scripts for the snRNA-seq downstream analyses were provided (Additional file 2).

### Analysis of FAP subtypes

Cells of FAPs were extracted in silico by the subset function of Seurat and regrouped into 5 subtypes. Annotation of each subtype was based on its characteristic genes and previous reports about their corresponding biological functions. The proportions of each subtype were also calculated [24, 25, 31].

### Pseudotime analysis by Monocle 3

The Seurat object of FAPs was extracted in silico and imported into the R package of Monocle 3 using the DietSeurat and as.cell_data_set functions. Subsequently, functions of cluster_cells, learn_graph, and order_cells were employed to construct the pseudotime trajectory. The dynamic expression levels of adipogenic and fibrogenic-related genes were visualized using the plot_genes_in_pseudotime function.

### Gene Ontology and Gene Set Enrichment Analysis

Gene Ontology (GO) and Gene Set Enrichment Analysis (GSEA) were performed with the R package, clusterProfiler v4.12.6. Terms were enriched with the nominal *P* value < 0.05 and false discovery rate *q* value < 0.05.

### RNA velocity analysis

The RNA velocity analysis was conducted using the Python packages velocyto and scVelo [30]. Initially, the filtered_barcodes.tsv files generated by CellRanger were used to create the overall loom files. Subsequently, the annotations of FAP subtypes and cell barcoding information from Seurat were extracted to filter the loom information generated in the first step. The scv.tl.velocity and scv.tl.velocity_embedding_stream functions from the scVelo package were employed to calculate the RNA velocity of the cell populations and to plot their developmental trends.

### In vitro culture of DF-1 cells

The DF-1 cell line (SNL-006; chicken embryo fibroblast) was sourced from Wuhan Sunncell Bio-Technology Co., Ltd. (Wuhan, China). DF-1 cells were cultured in normal growth medium, which consisted of DMEM (Gibco, Grand Island, USA) supplemented with 10% FBS (TransSerum, Nanjing, China) and 1% Antibiotic–Antimycotic (Gibco, Grand Island, USA). Adipogenesis was induced using a medium formulated following a previously established protocol [32], with slight modifications. The induction medium consisted of DMEM supplemented with 5% chicken serum (CS, Solarbio Science, Beijing, China), 0.5% fatty acid-albumin mixture (Linoleic Acid-Oleic Acid-Albumin, Sigma-Aldrich, St. Louis, USA), 5 µg/mL insulin (Sigma-Aldrich, Livonia, MI, USA), and 50 µmol/L all-*trans* retinoic acid (Sigma-Aldrich, St. Louis, USA). Medium was refreshed every 2 d.

Transfection of the control plasmid of pcDNA3.1 and the overexpression plasmid of pcDNA3.1-BMPER (GenePharma, Shanghai, China) was performed following the manufacturer's instructions of the Lipofectamine 3000 transfection reagent (Invitrogen, Carlsbad, CA, USA). After 6 h of transfection, the medium was replaced with normal growth medium. At 24 h post-transfection, the cells were treated with the adipogenic induction medium for 24 h before samples were stained for Oil Red O staining kit and for mRNA extraction.

### Cross-Species analysis of publicly available snRNA-seq datasets for human and mouse skeletal muscle cells

The snRNA-seq dataset for human and mouse skeletal muscle cells were obtained from the Human Skeletal Muscle Aging Atlas (https://www.muscleageingcellatlas.org) [33] and UCSC Cell Browser (https://cells.ucsc.edu/) [34]. The UMAP and feature plots generated in this study were modified from the original figures provided in the above websites. The original sequencing data was also published and accessible to the public by the authors [33, 35].

### Statistical analysis

For snRNA-seq, the analysis of differential expressed genes was performed by the default Wilcoxon Rank Sum test using the R package Seurat (v5.3.0). The other data were analyzed with GraphPad Prism v8.0 (La Jolla, CA, USA) and presented as means ± SEM (*n* = 8 for in vivo experiments and *n* = 5 for cell culture experiments). For multiple comparisons, the significance of differences between groups was assessed using one-way analysis of variance (ANOVA) followed by Tukey’s HSD test. For comparison between two groups, a two tailed *t*-test was applied. *P* values less than 0.05 were considered statistically significant.

## Results

### Dynamic changes of intramuscular fat deposition and FAP cellularity in Langshan chickens during growth

**Fig. 1:**
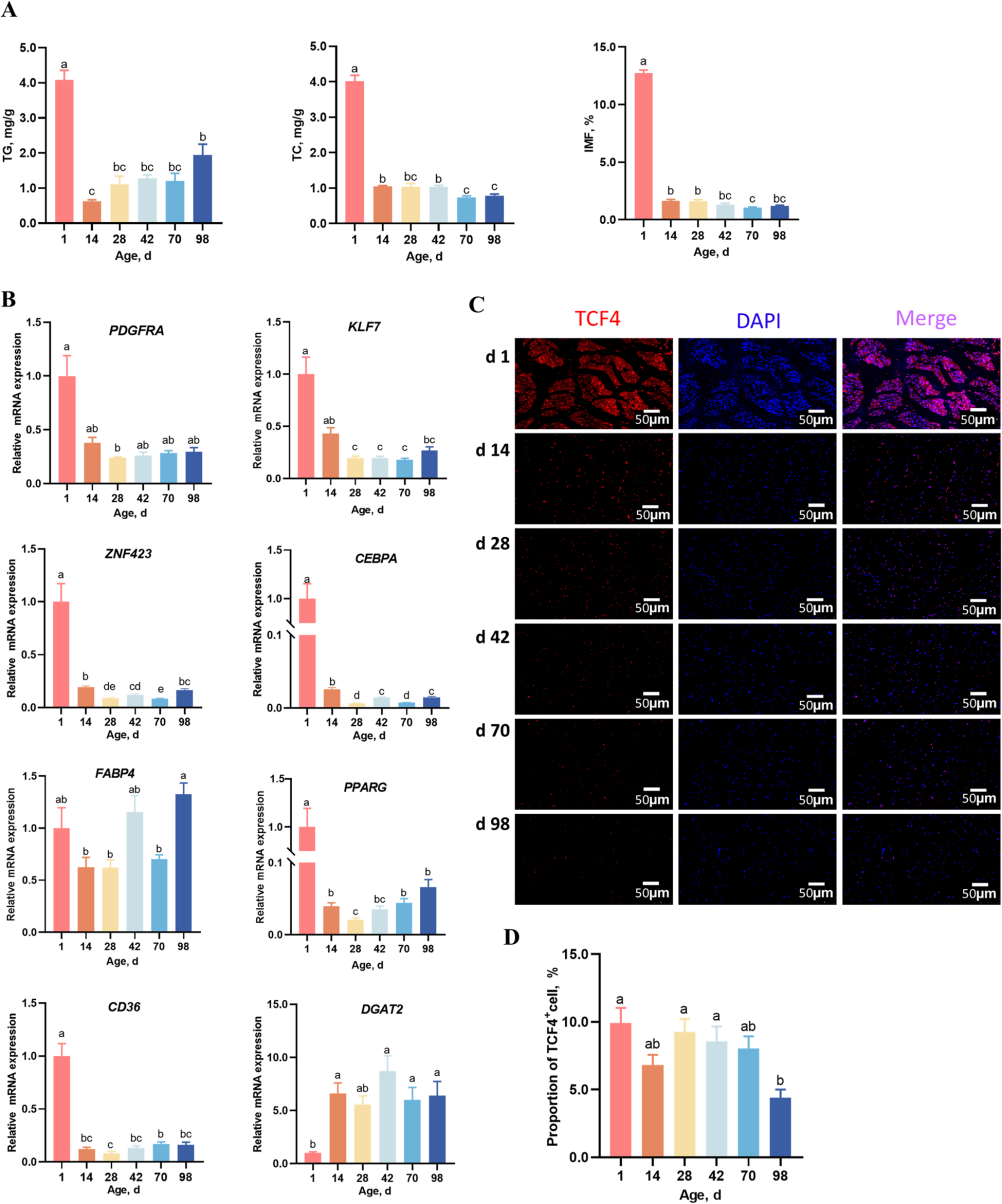
Dynamic changes of intramuscular fat deposition, adipogenesis-related gene expression, and TCF4^+^ cell numbers in chicken pectoralis major muscle across different growth stages. **A** Contents of triglyceride (TG), cholesterol (TC), and intramuscular fat (IMF). **B** mRNA expression of adipogenesis-related genes. **C** Representative immunofluorescence of TCF4^+^ fibro/adipogenic progenitors (FAPs) in the pectoral muscle. **D** Quantification of TCF4^+^ FAPs per unit area (1 mm^2^) of muscle tissue. All data are presented as mean ± SEM (*n* = 8 for A and B, *n* = 6 for C and D). Significant differences are denoted by different letters (*P* < 0.05)

The contents of TG, TC, and IMF in the pectoralis major muscle of Langshan chickens were measured at d 1, d 14, d 28, d 42, d 70, and d 98. As shown in Fig. 1A, the contents of TG, TC, and IMF were all highest at d 1. TG levels dropped sharply to their lowest at d 14, and then increased with age, with a statistically significant elevation at d 98. The TC contents remained unchanged (*P* > 0.05) from d 14 to d 42 until reaching their lowest at d 70 and d 98. The contents of the IMF remained relatively stable after d 1, and their lowest value was observed at d 70.

The expression levels of genes related to the adipogenic progression of FAPs were also detected (Fig. 1B). Consistent with the highest deposition of TG, TC, and IMF at d 1, adipogenesis-related genes including *PDGFRA*, *KLF7*, *ZNF423*, *CEBPA*, and *PPARG* expressed their highest levels at d 1 and declined until d 28. The expression of *PDGFRA* and *KLF7* remained relatively stable from d 28 to d 98, while significant elevations were observed for *ZNF423*, *CEBPA*, *FABP4* and *PPARG* at d 98 compared to those at d 28. In addition, the expression of the fatty acid uptake gene, *CD36*, also peaked at d 1 and reached its lowest level at d 28, while the expression of triglyceride synthesis-related gene, *DGAT2*, increased dramatically after d 1 and remained stable (*P* > 0.05) in subsequent stages. In addition, we observed the cell number changes of FAPs in the pectoralis major muscle (Fig. 1C). As a validated antibody for FAPs was still missing, we used a TCF4 antibody to label the fibrotic subpopulation of FAPs by immunofluorescence staining. The data showed that TCF4^+^ FAPs remained largely stable between d 1 to d 70, but there was a significant decrease at d 98 compared to those at d 1, d 28, and d 42. In summary, the above data suggests that the early postnatal life is a critical period for the proliferation and adipogenic transition of FAPs in Langshan chickens, while the TG synthesis and lipid deposition occurs at later lives.

### snRNA-seq analysis identified the population of FAPs in Langshan chicken muscles

**Fig. 2:**
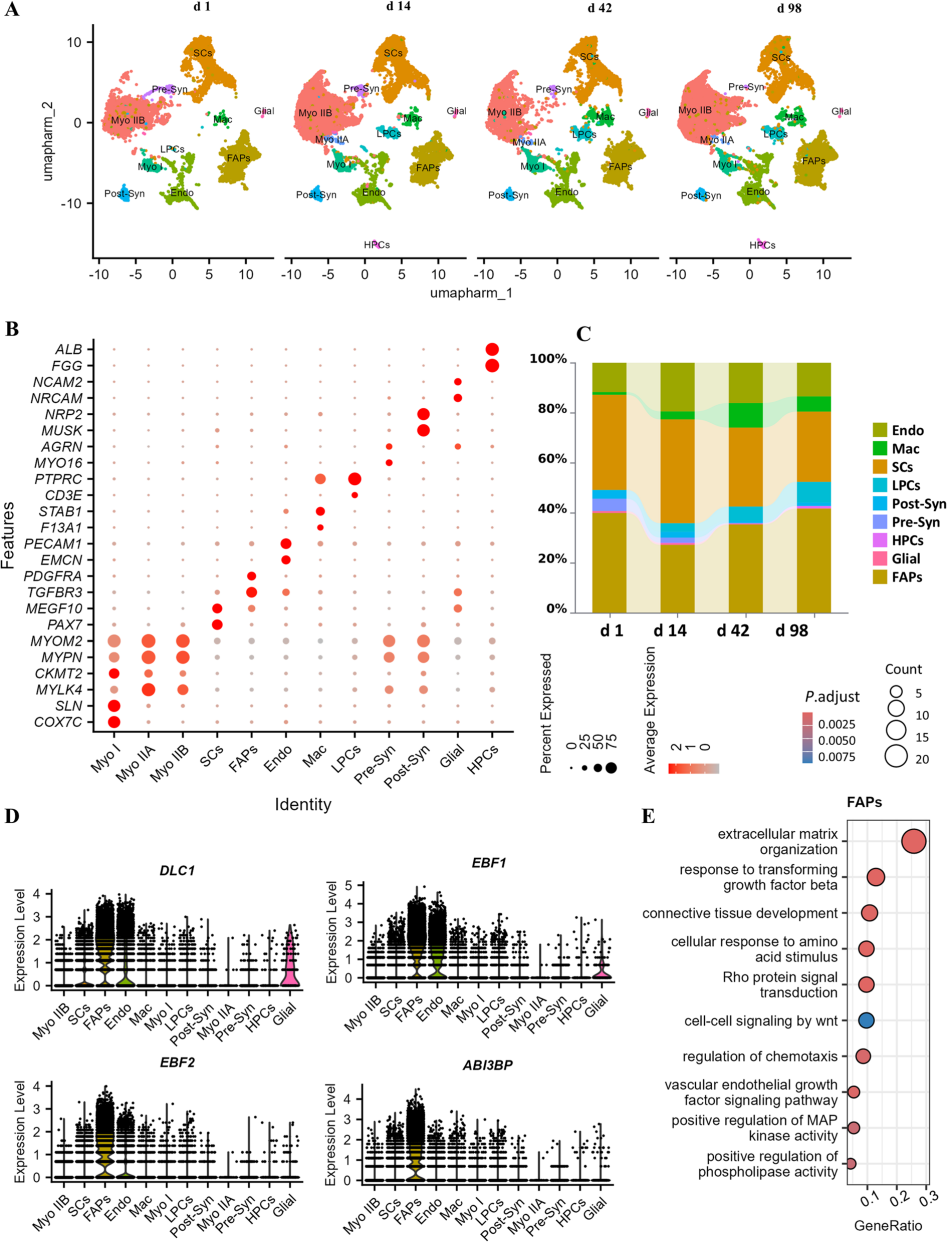
The cellular composition of Langshan chicken pectoralis major muscle at different ages (d 1, d 14, d 42, and d 98) revealed by the analysis of snRNA-seq. **A** UMAP reveals the distribution of major cell types. HPCs, hepatocytes (contaminated). **B** Dotplot shows representative marker gene expressions for each cell type. **C** Ratio of each cell type (myonuclei were excluded from the calculation). **D** Vlnplot shows adipogenesis-related characteristic gene expression for fibro/adipogenic progenitors (FAPs). **E** Gene Ontology (GO) enrichment analysis of top 100 marker genes for FAPs

To gain a comprehensive single-cell resolution of the pectoralis major muscle in Langshan chickens, snRNA-seq were performed at d 1, d 14, d 42, and d 98. A total of 53,542 cells passed quality control with a median gene number of 958. Using unsupervised uniform manifold approximation and projection (UMAP) analysis (Fig. 2A–E), we annotated 12 cell types including myonuclei type I (Myo I), Myo IIA, Myo IIB, satellite cells (SCs), FAPs, endothelial cells (Endo), macrophages (Mac), lymphocytes (LPCs), post-synaptic cells (Post-Syn), pre-synaptic cells (Pre-Syn), hepatocytes (HPCs), and glial cells (Glial). All these cell types, except Myo IIA, LPCs and HPCs, exhibited consistent distribution across four developmental stages, indicating unbiased sampling (Fig. 2A). Expression of 2 characteristic marker genes for each cell type were shown on Fig. 2B. FAPs in Langshan chicken muscles also express unique and high levels of *PDGFRA* and *TGFBR3* as observed in other species. The proportion of FAPs among mesenchymal cells declined from 39.90% at d 1 to 27.13% at d 14, followed by a gradual increase to 41.65% at d 98 (Fig. 2C).

In addition to well-recognized marker genes, we observed unique high expressions of genes with potential adipogenic regulatory functions, including *DLC1*, *EBF1*, *EBF2*, and *ABI3BP*, in the FAP population (Fig. 2D). Moreover, GO analysis of the top 100 marker genes for FAPs were conducted (Fig. 2E). The enriched biological terms of “extracellular matrix organization”, “response to transforming growth factor beta”, “connective tissue development”, and “positive regulation of phospholipase activity” further confirmed the fibrotic and adipogenic potentials of FAPs. Additionally, FAPs were shown to be regulated by amino acid stimulus and signaling pathways of Rho, WNT, VEGFR and MAPK. Therefore, our snRNA-seq analysis successfully identified the FAP population, revealed their dynamic changes during growth, and uncovered novel marker genes and signaling pathways involved in their maintenance, providing valuable insights for further analysis of their adipogenic regulatory mechanisms.

### Chicken fibro/adipogenic progenitors are a heterogeneous population

**Fig. 3:**
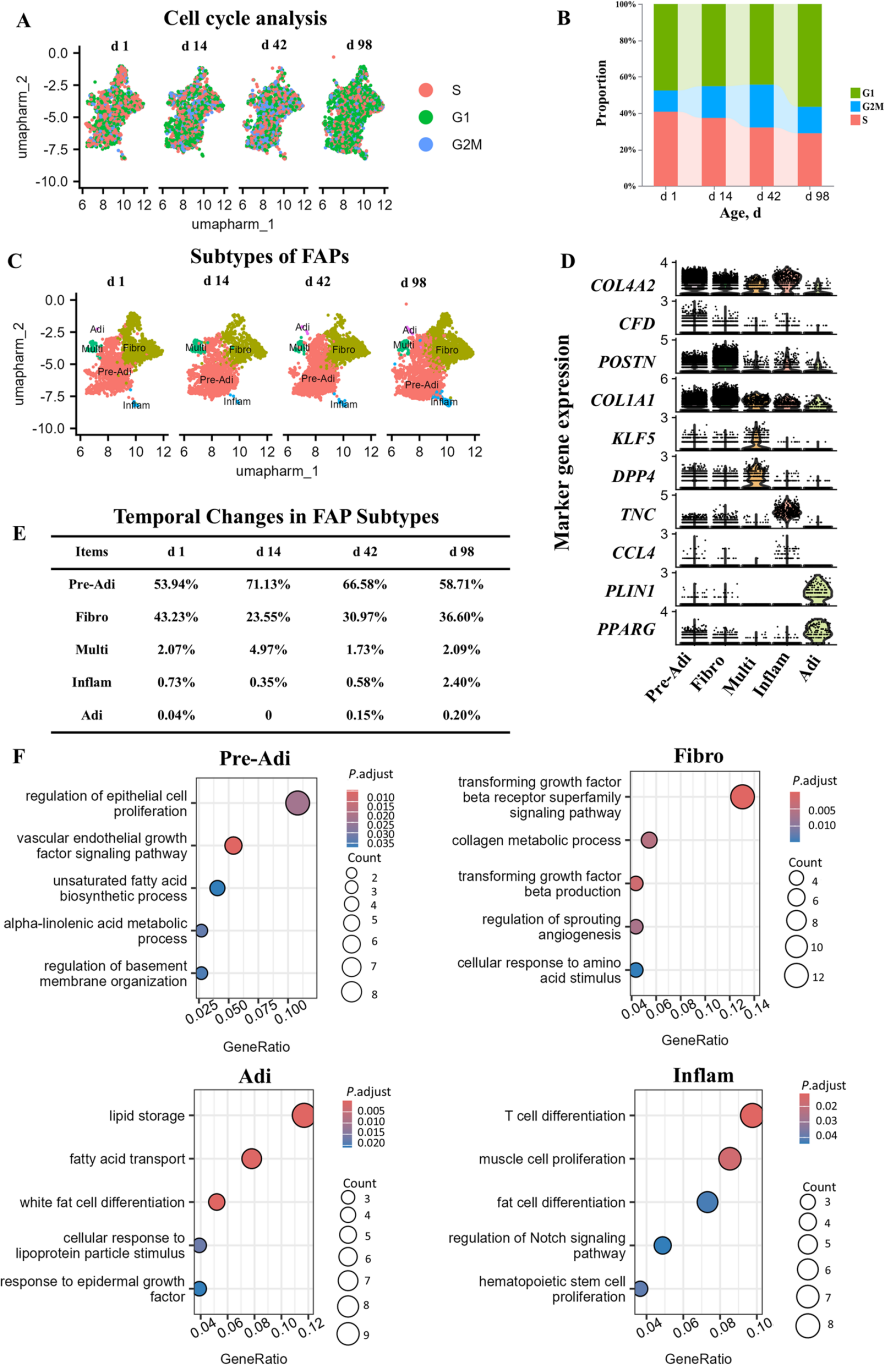
Characteristics of subtypes of fibro/adipogenic progenitors (FAPs). **A** Cell cycle analysis of FAPs. **B** Proportion of FAPs in G1, G2M, and S stages of cell cycle. **C** Identification of FAP subpopulations. **D** Marker genes for each FAP subtype. **E** Proportions of each FAP subtype. **F** Enriched GO terms of biological process for top 100 marker genes of each FAPs subtype

To further understand the composition and characteristics of FAPs in chickens, we subset the population of FAPs in silico and conducted analysis of cell cycle, subtype regrouping, and GO pathways (Fig. 3A–F). As shown in Fig. 3A and B, FAPs at d 98 exhibited the highest percentage (56.44%) of cells in the G1 phase and the lowest percentage (29.02%) in the S phase, suggesting reduced proliferation but enhanced differentiation potential of FAPs at d 98 compared to the other time points.

The FAPs were regrouped into 5 clusters, which were annotated as multi-potential subtype (Multi), pre-adipogenic subtype (Pre-Adi), adipogenic subtype (Adi), fibrotic subtype (Fibro), and inflammatory subtype (Inflam) based on their marker gene expressions (Fig. 3C and D). Analysis of the proportion of each subtype showed that the Pre-Adi subtype reached its highest ratio at d 14, then steadily decreased until d 98 (Fig. 3E). In contrast, the Adi subtype exhibited the opposite pattern. Consistent with the significant increase in pectoral muscle triglyceride content from d 14 to d 98, the concurrent decrease in pre-adipocyte proportion and slight increase in adipocyte proportion align with potential ongoing differentiation processes; we therefore speculate that differentiation toward mature adipocytes may have occurred during this time period. Meanwhile, the proportion of the Fibro subtype peaked at d 1, dropped to its lowest at d 14, and then rose steadily until d 98. Additionally, the proportion of the Inflam subtype increased 6.9-fold at d 98 compared to that at d 14. The characteristics of Pre-Adi, Adi, Fibro, and Inflam subtypes were further validated by GO pathways as shown in Fig. 3F. Interestingly, the terms enriched for the Inflam subtypes suggested their potential functions for the regulation of “muscle cell proliferation” and “fat cell differentiation”. The resolution of FAP compositions especially the identification of Pre-Adi and Adi subtypes provided a novel opportunity to further understand the adipogenic progression of FAPs in chicken muscles during growth.

### Analysis of RNA velocity and pseudo-trajectory reveals fate determination differences of FAPs in chicken muscles during growth

**Fig. 4:**
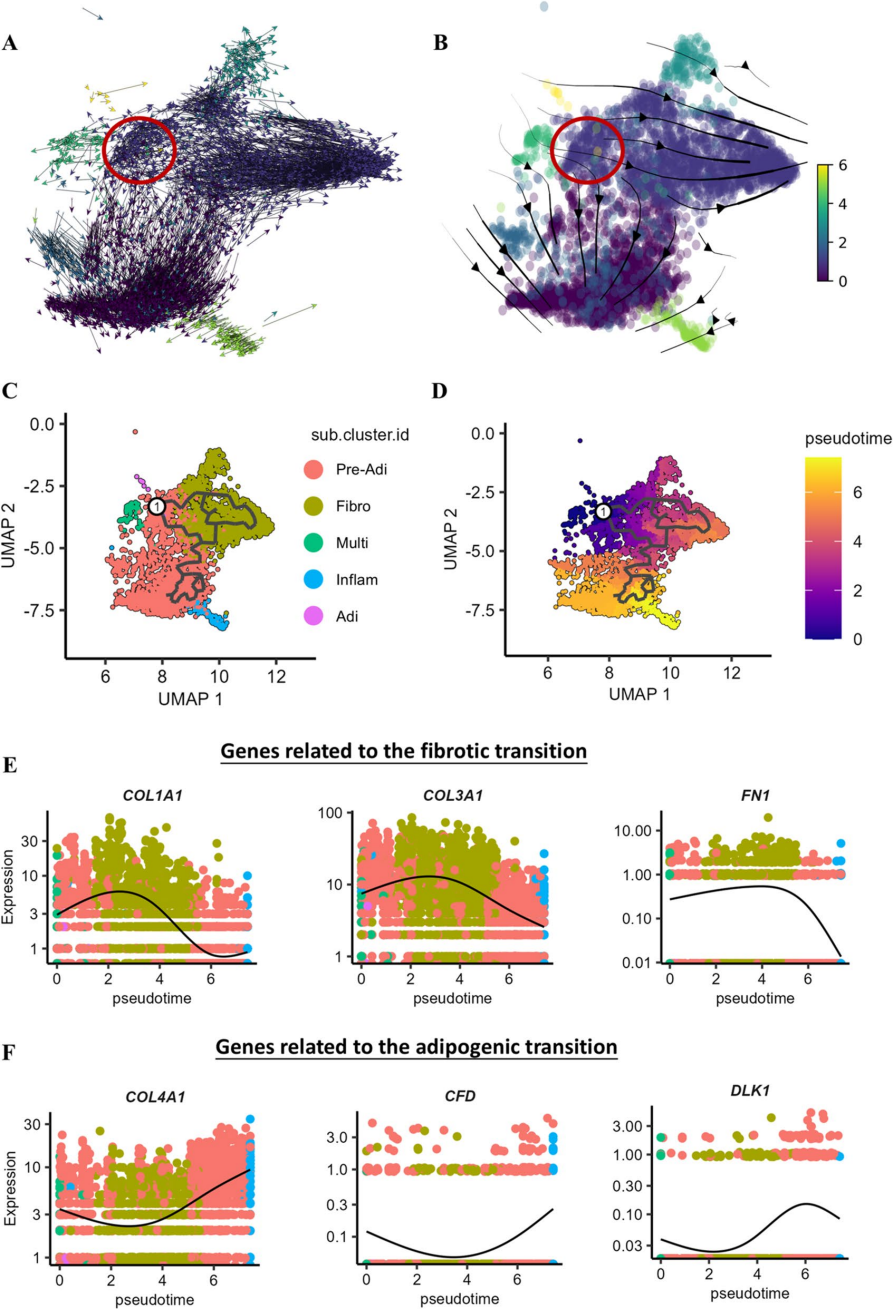
RNA velocity and pseudo-trajectory analysis of FAP subpopulation. The directions of arrows (**A**) and streamlines (**B**) indicate developmental paths of FAPs by RNA velocity analysis. Pseudo-trajectory predicted by Monocle 3 was illustrated by cell cluster distribution (**C**) and pseudotime (**D**). Dynamic expression changes of fibrotic (**E**) and adipogenic (**F**) transition-related genes are shown

The RNA velocity analysis was performed on FAPs from the d 98 Langshan chicken muscles. The arrows (Fig. 4A) and streamlines (Fig. 4B) illustrated the developmental potential of FAPs from less mature to more mature states. Consistent directional patterns were also observed in FAPs from earlier time points (d 1, d 14, d 42; Fig. S1C in Additional file 1). The results predicted two distinct developmental directions from the same origin (labeled in a red circle in Fig. 4A and B, and Fig. S1 in Additional file 1). Consistently, pseudo-trajectory analysis by Monocle 3 revealed a similar development pattern of FAPs (Fig. 4C and D). The first developmental path mainly involved the Fibro subtype, indicating cell state changes during the fibrotic progression of FAPs. The second developmental path predominantly progressed through the Adi subtype, reflecting cell state changes during adipogenic transition of FAPs.

Next, we checked the expression of genes involved in the fibrotic and adipogenic transitions of FAPs across the pseudo-trajectory path (Fig. 4E and F). The expression of fibrotic marker genes (*COL1A1*, *COL3A1*, and *FN1*) progressively decreased along pseudotime, demonstrating a gradual decline in their fibrotic potential (Fig. 4E). Conversely, adipogenic transition-related genes (*COL4A1*, *CFD*, and *DLK1*) showed elevated expression levels over pseudotime, indicating enhanced adipogenic differentiation capacity in FAPs (Fig. 4F). Similar expression patterns of above genes were also observed in the pseudo-trajectory path constructed using combined samples from all-time points (Fig. S1A and S1B in Additional file 1). Collectively, the above data demonstrated that FAPs initially exhibited higher fibrotic capacity, which progressively declines over time, while their adipogenic capacity was enhanced during developmental progression.

### The adipogenic transition of FAPs requires elevated expression of BMPER

**Fig. 5:**
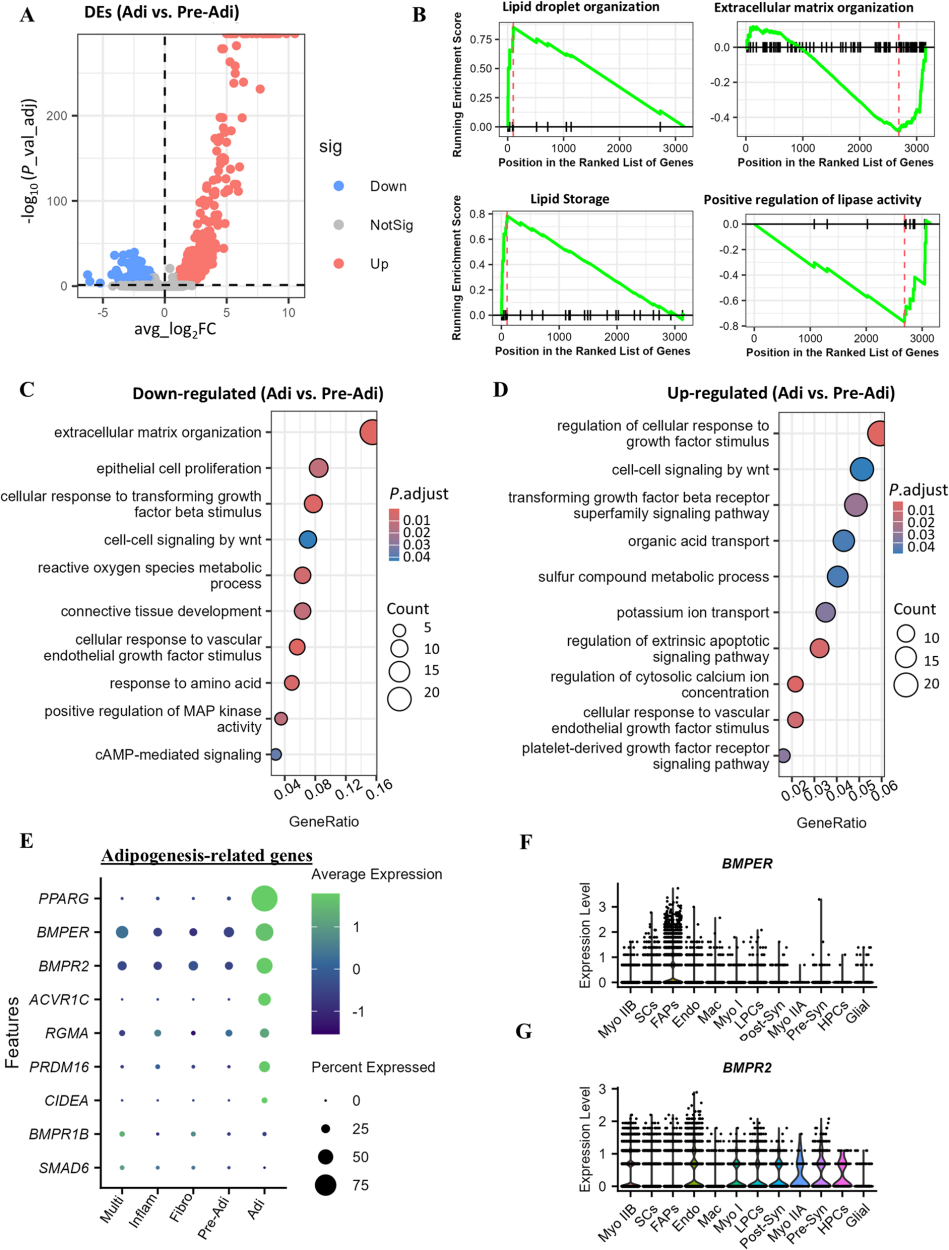
Comparative analysis of the transcriptomes of adipogenic (Adi) to pre-adipogenic (Pre-Adi) subtypes of FAPs. **A** Volcano plot shows the differentially expressed genes (DEGs, |avg_log_2_FC| > 0.8, adj_*P* < 0.05). **B–D** Enriched GSEA (**B**), down-regulated (**C**) and up-regulated (**D**) GO terms of biological process based on DEGs. **E** Dotplot shows expression levels of adipogenesis-related genes from the above enriched terms of “transforming growth factor beta receptor superfamily signaling pathway”. **F** and **G** Distribution of *BMPER* and *BMPR2* expressions among the major cell types in the pectoralis major muscle

To better understand the regulatory mechanisms governing adipogenic progression in FAPs, we compared gene expression between Adi and Pre-Adi subtypes. As shown in Fig. 5A, significantly more genes were up-regulated (479) than down-regulated (184) in the Adi subtype compared to the Pre-Adi subtype. GSEA analysis revealed that the biological processes of “lipid droplet organization” and “lipid storage” were enhanced in the Adi subtype, whereas “extracellular matrix organization” and “positive regulation of lipase activity” were decreased, indicating greater maturity of Adi subtype in adipogenic progression of FAPs (Fig. 5B).

GO analysis of the differential expressed genes (DEGs) further identified important signaling pathways for this adipogenic progress (Fig. 5C and D). Signaling pathways related to TGFB stimulus, MAPK, and cAMP were uniquely down-regulated in the Adi subtype, while biological process of “transforming growth factor beta receptor superfamily signaling pathways”, “sulfur compound metabolic process”, “platelet-derived growth factor receptor signaling pathway”, “regulation of cytosolic calcium ion concentration” were enhanced. We further checked the expression of adipogenesis-related genes from the enriched GO term of “transforming growth factor beta receptor superfamily signaling pathways” across different FAP subtypes (Fig. 5E). The top 3 expressed genes were involved in the BMP signaling pathway including *PPARG*, *BMPER*, and *BMPR2*. Moreover, the distribution of *BMPER* was found to be highly specific for FAPs compared to other cell types in chicken muscles, while *BMPR2* was more broadly distributed across cell types (Fig. 5F and G). Therefore, our data found that BMPER not only serves as a marker for FAPs but may also play important roles in promoting their adipogenic progression.

### Transcriptomic differences between FAPs collected on d 1 and d 14

**Fig. 6:**
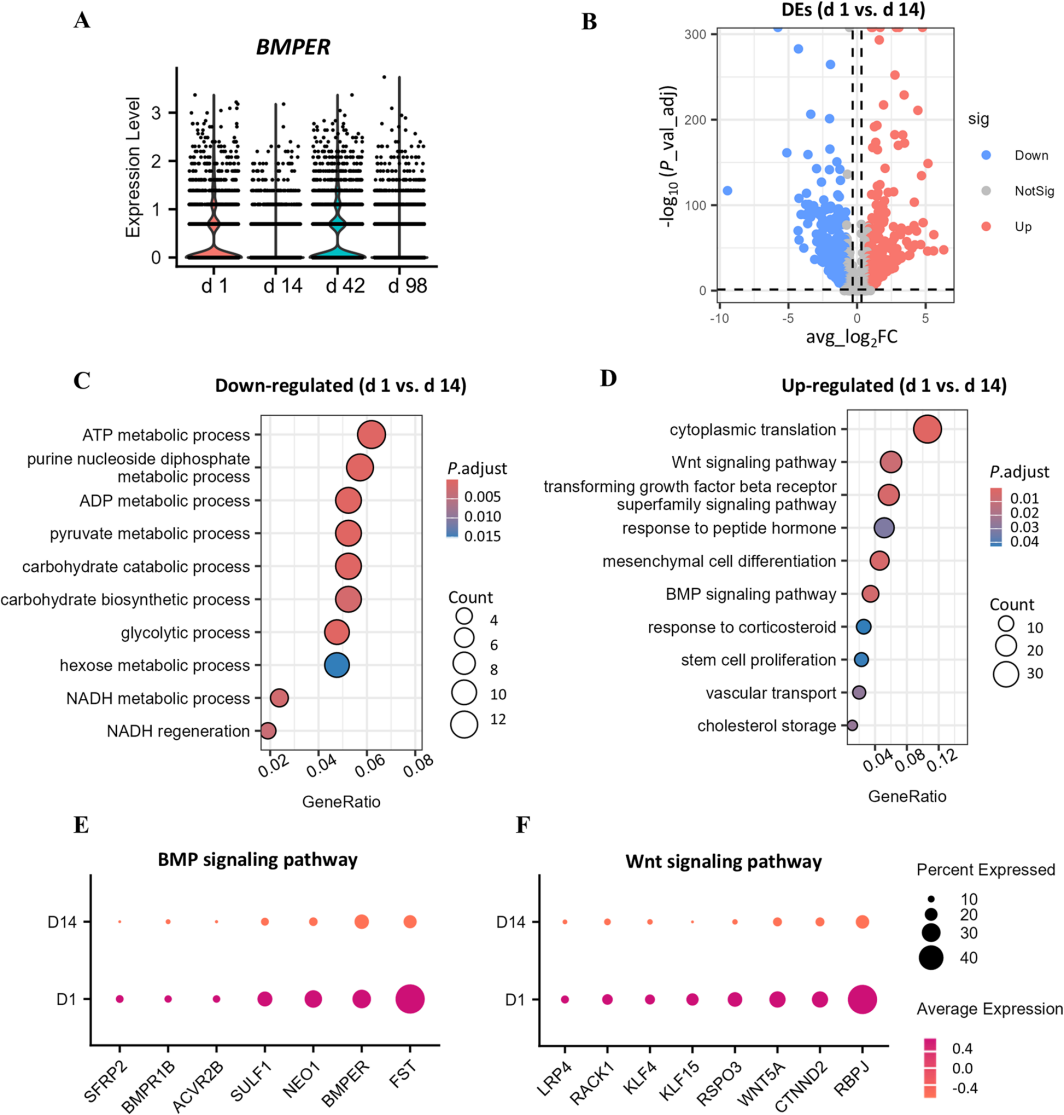
Comparative analysis of the transcriptomes of FAPs at d 1 to those at d 14. **A** Expression levels of *BMPER* in FAPs from different ages. **B** Volcano plot shows differentially expressed genes (DEGs, |avg_log_2_FC| > 0.8, adj_*P* < 0.05) in FAPs. **C** and **D** Enriched down-regulated (**C**) and up-regulated (**D**) GO terms of biological process based on DEGs. **E** and **F** Dotplot shows expression levels of genes from the above enriched terms of BMP (**E**) and Wnt (**F**) signaling pathways

As the lipid deposition was significant higher in muscle samples on d 1 compared to d 14 (Fig. 1A), we compared gene expression profiles of FAPs at these two time points. Interestingly, *BMPER* expression was significantly reduced on d 14 compared to d 1 (*P* < 0.001, Fig. 6A). Transcriptomic comparison revealed 718 upregulated genes and 529 downregulated genes in d 1 FAPs (Fig. 6B). GO analysis of the down-regulated DEGs revealed reduced metabolic activity for ATP, ADP, NADH, pyruvate, and carbohydrate in d 1 FAPs (Fig. 6C). Conversely, terms of “cholesterol storage” were enriched in up-regulated pathways, suggesting stronger lipid deposition ability in d 1 FAPs (Fig. 6D). Terms of “cytoplasmic translation”, “mesenchymal cell differentiation”, and “stem cell proliferation” indicated that FAPs were more active at d 1. In addition, important adipogenesis-related signaling pathways were identified such as “Wnt signaling pathway”, “transforming growth factor beta receptor superfamily signaling pathway”, and “BMP signaling pathway”. The genes involved in the BMP signaling comprised *FST*, *BMPER*, *NEO1*, *SULF1*, *ACVR2B*, *BMPR1B* and *SFRP2* (Fig. 6E), while Wnt signaling pathway-related genes included *RBPJ*, *CTNND2*, *WNT5A*, *RSPO3*, *KLF15*, *KLF4*, *RACK1*, and *LRP4 (*Fig. 6F). Therefore, the function of BMPER in adipogenic progression of FAPs warrants further investigation.

### BMPER induced adipogenic differentiation of DF-1 cell lines in vitro

**Fig. 7:**
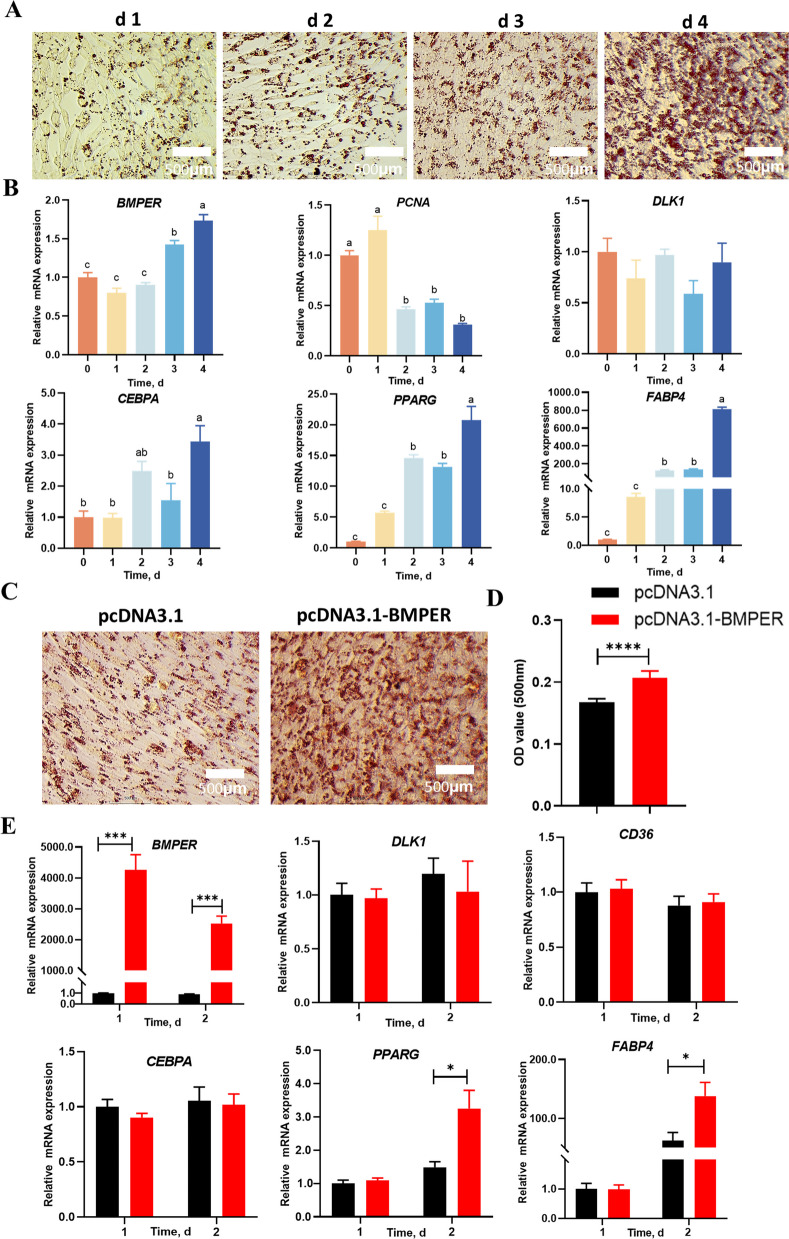
Functional studies of BMPER for adipogenic differentiation of DF-1 cells in vitro. **A** Oil Red O staining shows the process of adipogenic differentiation of DF-1 cells. **B** Relative mRNA expression of key adipogenesis-related genes during the differentiation process of DF-1 cells. **C** Representative Oil Red O staining of DF-1 cells after transfection with pcDNA or pcDNA-BMPER plasmids. **D** Quantification of lipid accumulation by optical density. **E** Expression of adipogenesis-related marker genes in control versus BMPER-overexpressing DF-1 cells during differentiation. All data are presented as mean ± SEM (*n* = 5). For B, significant differences are denoted by different letters (*P* < 0.05). For panels D and E, ^*^P < 0.05, ^***^P < 0.001, ^****^P < 0.0001

Chicken embryonic cell line DF-1 demonstrated adipogenic and fibrotic differentiation capacities comparable to those of FAPs [32]. This similarity establishes DF-1 cells as a valuable experimental model for investigating the molecular regulatory mechanisms of FAP fate determination. As shown in Fig. 7A, DF-1 cells were induced for adipogenic differentiation for 4 d in vitro. The gene expression of *BMPER* was found to be significantly enhanced at d 3 and continued to increase at d 4 (Fig. 7B). *PCNA* expression reached its highest level at d 1, and subsequently decreased, while no difference was found in *DLK1* expression throughout the culture process. Adipogenic differentiation marker genes of *CEBPA*, *PPARG*, and *FABP4* exhibited similar trends of elevated expression over time. Notably, *CEBPA*, *PPARG*, and *FABP4* showed pronounced elevation at d 4 compared to d 3, demonstrating a temporal delay of 1 d relative to BMPER activation.

Furthermore, we transfected the DF-1 cells with a pcDNA3.1-BMPER plasmid to overexpress BMPER. At d 2, the Oil RedO staining (Fig. 7C and D) showed significantly enhanced lipid formation in the pcDNA-BMPER group compared to the pcDNA3.1 group (the control group). The gene expression of *BMPER* at d 1 and d 2 was dramatically elevated, which was accompanied by significantly increased expression of *PPARG* and *FABP4* at d 2 (Fig. 7E). No differences were found for the expression of *DLK1*, *CD36*, and *CEBPA* throughout the culture process. In summary, the above data verified the function of BMPER for the adipogenic maturation of DF-1 cells in vitro.

### Cross species conservation of unique BMPER expression in FAPs

**Fig. 8:**
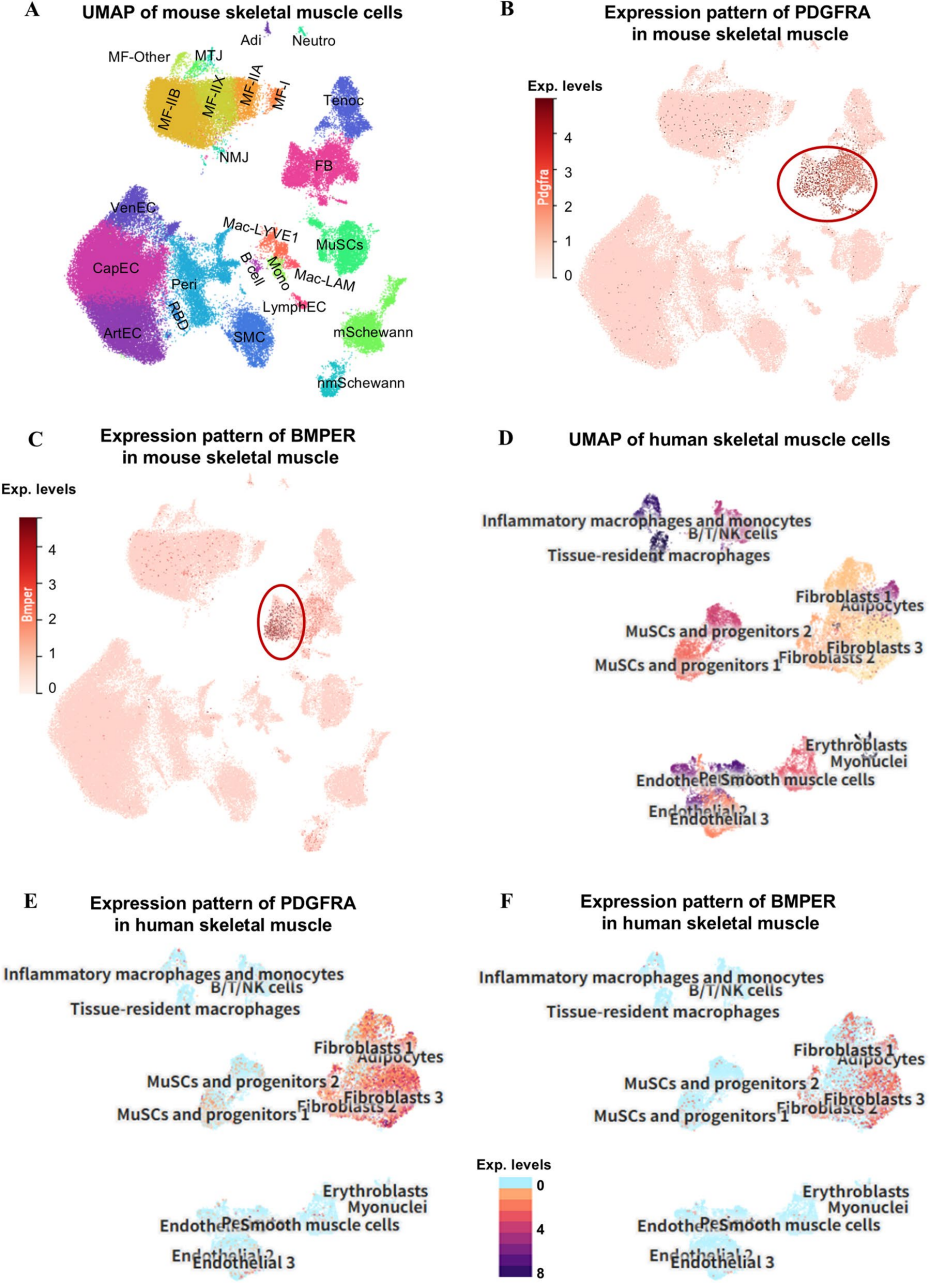
Cross-species conservation of BMPER expression in FAPs of the skeletal muscle. For (**A**–**C**), figures were modified from the interactive tools of a publicly accessible dataset for mouse skeletal muscle (https://www.muscleageingcellatlas.org). **A** UMAP shows cell type composition of the mouse skeletal muscle. **B** and **C** Featureplot shows the distribution of PDGFRA and BMPER expressions in mouse skeletal muscle. For (**D–F**), figures were modified from the interactive tools of a publicly accessible dataset for human skeletal muscle (https://cells.ucsc.edu/). **D** UMAP shows cell type composition of the human skeletal muscle. **E** and **F** Featureplot shows the distribution of PDGFRA and BMPER expressions in human skeletal muscle

To elucidate the evolutionary conservation of BMPER expression in FAPs, we analyzed its distribution in mouse (Fig. 8A–C) and human (Fig. 8D–F) skeletal muscle using two publicly accessible datasets with interactive web tools: Muscle Ageing Cell Atlas and UCSC Cell Browser. As shown in Fig. 8A and B, the expression of PDGFRA was uniquely found in the population of fibroblast (FB, comparable annotation as FAPs) in the mouse skeletal muscle. The expression of BMPER was more concentrated in a portion of PDGFRA-expressing FBs (labelled in a red circle). In the human skeletal muscle, the PDGFRA-expressing cells were divided into 4 subtypes including Fibroblast 1–3 and Adipocytes (Fig. 8D and E). Consistently, the expression of BMPER was specifically found within a portion of these cells, especially *DCN*/*GCN/MYOC* + fibroblast 2 and *APOD/CFD/PLAC9* + adipocytes. Therefore, the expression of BMPER was highly conserved among humans, mouse and chicken skeletal muscle.

## Discussion

Recent studies have established FAPs as the principal cellular source of intramuscular adipocytes in mammals [6, 7]. However, our understanding of the characteristics of FAPs and their regulatory mechanism of adipogenic progression in farm animals, especially in poultry, remains largely unexplored. Here, utilizing snRNA-seq analysis, we constructed the first comprehensive developmental atlas of chicken skeletal muscle FAPs spanning their growth period.

Notably, five FAP subpopulations identified in this study exhibit clear functional correspondence to heterogeneous populations reported across mammalian species. The CFD-marked adipogenic subset resembles adipogenic precursors in bovine skeletal muscle [24]. The COL1A1-high fibrogenic subset parallels subpopulations of fibroblast-like cells in bovine skeletal muscle and human muscle [24, 35]. The inflammation-featured subpopulation is comparable to CCL2^+^ FAPs described in human muscle [35]. In addition, breeds with higher intramuscular fat deposition possess more FAPs in numbers and with greater adipogenic potential [24, 36]. This cross-species subpopulation correspondence suggests that intramuscular adipogenesis may follow a conserved cellular logic.

Building upon these subpopulation identities, we demonstrate that chicken FAPs undergo a temporally coordinated transition from a fibrogenic state toward adipogenic commitment, characterized by reciprocal shifts in the proportions of preadipocyte (Pre-Adi) and fibrogenic (Fibro) subsets. This process mirrors the fibro-adipogenic fate plasticity observed in mammalian muscle yet occurs within a compressed developmental window adapted to the rapid growth demands of chickens [24]. Pseudotime analysis further revealed that chicken FAPs initially exhibit high fibrogenic capacity (e.g., elevated COL1A1 expression), which gradually declines throughout development; concomitantly, adipogenic competence progressively intensifies. This "fibrosis-first, adipogenesis-later" developmental trajectory suggests that the initial fibrogenic capacity of neonatal FAPs represents a "default" mesenchymal state that undergoes bidirectional regulation during cell fate decisions: one subset acquires adipogenic potential (Pre-Adi lineage), while another persists in extracellular matrix remodeling (Fibro lineage). The biological significance of this ordered transition presumably lies in ensuring that adipogenesis initiates only after appropriate extracellular matrix scaffolding has been established, thereby precisely balancing early muscle growth against later fat infiltration within this compressed developmental timeframe—as evidenced at the tissue phenotypic level by the significant increase in pectoral muscle TG content from d 14 to d 98, reflecting the dominant adipogenic capacity during later developmental stages.

Through comparative transcriptomic analysis of adipocyte and preadipocyte subtypes, we identified BMPER as a specific marker of mature adipocyte subsets and a critical driver of adipogenic differentiation. BMPER, a context-dependent regulator of BMP signaling [37–39], has been established in mammals [40, 41], yet its cell-specific function in avian skeletal muscle FAPs remains uncharacterized. We demonstrate that BMPER is specifically enriched in mature adipocyte subsets, and in vitro experiments confirmed that BMPER overexpression significantly activates PPARG and FABP4 while promoting lipid accumulation, establishing its functional role as an initiator of adipogenesis. Importantly, we also detected conserved BMPER expression in human and murine skeletal muscle FAPs, suggesting its universal role as a cross-species regulator of adipogenic differentiation.

We acknowledge several limitations of this study. First, due to the lack of commercially available antibodies against classic mammalian FAP markers (PDGFRA and SCA1) validated for avian species, we employed TCF4 for immunofluorescence staining. While TCF4 effectively labeled fibro-adipogenic progenitors in chicken muscle, future development of avian-specific FAP markers would enhance precision. Second, although our pseudotime analysis captured major developmental transitions, including earlier time points (e.g., d 3, d 7) would help greatly to dissect the precise molecular switches governing the fibrosis-to-adipogenesis transition. Finally, the correlation between BMPER expression and lipid deposition metrics across individual animals or time points needs further quantitative analysis.

## Conclusions

In summary, to our knowledge, this study presents the first comprehensive analysis of FAP composition and dynamics during chicken skeletal muscle growth, revealing a "fibrosis-to-adipogenesis" developmental paradigm and establishing BMPER as a critical driver of FAP adipogenic differentiation across species. These findings not only advance our understanding of cell fate determination mechanisms in skeletal muscle but also provide specific molecular targets for optimizing intramuscular fat deposition in poultry through early intervention in FAP subpopulation proportions, with potential applications for meat quality improvement. Furthermore, these insights offer a novel theoretical framework for understanding FAP fate determination under both physiological and pathological conditions.

## Supplementary Information

Additional file 1: Table S1. Feed ingredients and nutrient levels of basal diet. Fig. S1. Pseudo-trajectory inferred from integrated data across all time points, visualized by cell distributionand pseudotime. Dynamic expression patterns of genes associated with fibrotic or adipogenic transition along the pseudotime. RNA velocity analysis performed for individual time points. Arrows and streamlines indicate the predicted developmental paths of FAPs. Red circles denote regions with shared developmental patterns across time points.

Additional file 2: R and Python script.

## Data Availability

The raw sequence data reported in this paper have been deposited in the Genome Sequence Archive (Genomics, Proteomics & Bioinformatics 2025) in National Genomics Data Center (Nucleic Acids Res 2025), China National Center for Bioinformation/Beijing Institute of Genomics, Chinese Academy of Sciences (GSA: CRA032199) that are publicly accessible at https://ngdc.cncb.ac.cn/gsa.

## Abbreviations

BMPER: BMP-binding endothelial regulator
DEG: Differentially expressed gene
FAP: Fibro/adipogenic progenitor
GO: Gene Ontology
GSEA: Gene Set Enrichment Analysis
IMF: Intramuscular fat
scRNA-seq: Single-cell RNA sequencing
snRNA-seq: Single-nucleus RNA sequencing
TC: Total cholesterol
TG: Triglyceride
