# Supplementary material for: BMPER induces the adipogenic differentiation of fibro/adipogenic progenitors and promotes intramuscular fat deposition in chickens
Source: J Anim Sci Biotechnol. 2026 Apr 19;17:72. doi: 10.1186/s40104-026-01389-9 (PMC13092164; doi:10.1186/s40104-026-01389-9)
Supplement: Supplementary file 1 — Additional file 1: Table S1. Feed ingredients and nutrient levels of basal diet. Fig. S1. Pseudo-trajectory inferred from integrated data across all time points, visualized by cell distributionand pseudotime. Dynamic expression patterns of genes associated with fibrotic or adipogenic transition along the pseudotime. RNA velocity analysis performed for individual time points. Arrows and streamlines indicate the predicted developmental paths of FAPs. Red circles denote regions with shared developmental patterns across time points. [file 40104_2026_1389_MOESM1_ESM.docx]

**Supplementary material**

**Supplementary material 1**

**Table S1. Feed ingredients and nutrient levels of basal diet**.

| **Ingredient（%）** | **1-21 days of age** | **22-98 days of age** |
| --- | --- | --- |
| Corn | 60.00 | 63.54 |
| Soybean meal | 32.85 | 27.49 |
| Corn gluten meal (56%, CP) | 1.92 | 3.00 |
| Soybean oil | 1.46 | 2.03 |
| Dicalcium phosphate | 1.40 | 1.72 |
| Limestone | 1.26 | 1.24 |
| Salt | 0.30 | 0.32 |
| L-Lysine | 0.20 | 0.13 |
| DL-Methionine | 0.20 | 0.11 |
| Choline chloride (50%) | 0.19 | 0.20 |
| Vitamin-mineral premix^1^ | 0.22 | 0.22 |
| **Nutrient level^2^** |  |  |
| Metabolizable energy（MC/Kg） | 12.01 | 12.55 |
| Crude protein (%) | 20.00 | 19.00 |
| Calcium (%) | 1.00 | 0.90 |
| Total phosphorus (%) | 0.69 | 0.63 |
| Available phosphorus (%) | 0.45 | 0.40 |
| Lysine (%) | 1.05 | 1.00 |
| Methionine (%) | 0.48 | 0.43 |
| Threonine (%) | 0.74 | 0.76 |
| Tryptophan (%) | 0.22 | 0.23 |

^1^Premix provided per kilogram of feed: VA 9000 IU, VD_3_ 2700 IU, VE 20 mg, VB_1_ 3.0 mg, VK_3_ 2.4 mg,VB_2_ 6.4mg,VB_6_ 2.8 mg, VB_12_ 0.01 mg, D-pantothenic acid 11 mg, folic acid 0.70 mg, biotin 0.08 mg, niacin 40 mg, choline 460 mg, Mn 60 mg, Fe 40 mg, Cu 10 mg, Zn 55 mg, I 1.6 mg, Se 0.35 mg.

^2^Calculated values.


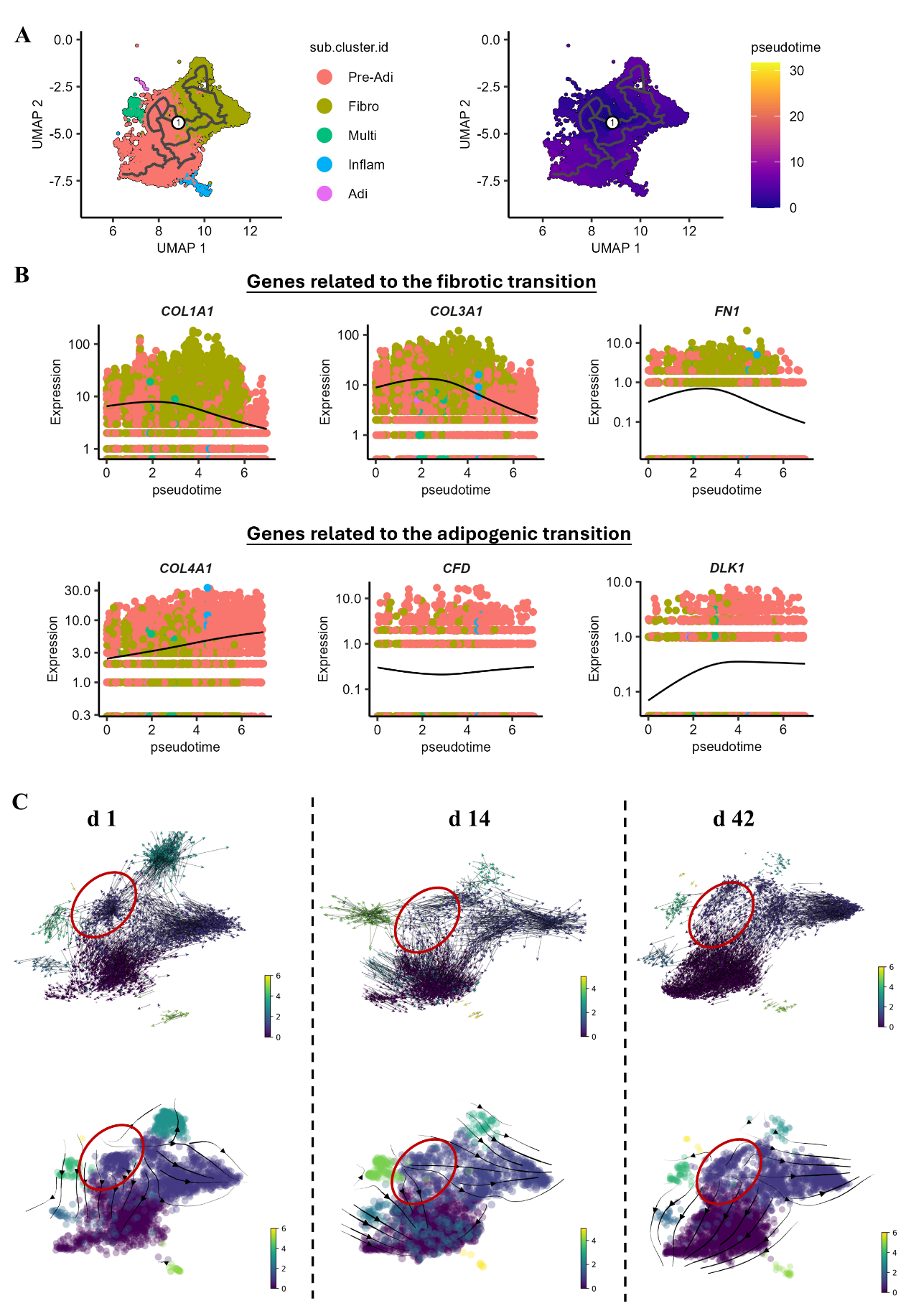


**Fig. S1.** (A) Pseudo-trajectory inferred from integrated data across all time points (Monocle 3), visualized by cell distribution (left) and pseudotime (right). (B) Dynamic expression patterns of genes associated with fibrotic or adipogenic transition along the pseudotime. (C) RNA velocity analysis performed for individual time points (d 1, d 14, and d 42). Arrows and streamlines indicate the predicted developmental paths of FAPs. Red circles denote regions with shared developmental patterns across time points.
