# Supplementary material for: BMPER induces the adipogenic differentiation of fibro/adipogenic progenitors and promotes intramuscular fat deposition in chickens
Source: J Anim Sci Biotechnol. 2026 Apr 19;17:72. doi: 10.1186/s40104-026-01389-9 (PMC13092164; doi:10.1186/s40104-026-01389-9)
Supplement: Supplementary file 2 — Additional file 2: R and Python script. [file 40104_2026_1389_MOESM2_ESM.docx]

**Supplementary material 2**

**R and python script**

**1. For Seurat package**

**# load necessary package**

library(Seurat)

library(ggplot2)

library(sctransform)

set.seed(123)

library(patchwork)

library(ComplexHeatmap)

library(scCustomize)

library(cowplot)

library(dplyr)

**# Combine data matrix of 4 samples**

LS_1.data <- Read10X(data.dir = "F:/For.Analysis/data/matrix/2405132_Ls1/filtered_feature_bc_matrix")

LS_1 <- CreateSeuratObject(counts = LS_1.data, project = "LS_1", min.cells = 3, min.features = 50)

LS_1@meta.data[,"treatment"]<-"LS"

LS_1@meta.data[,"day"]<-"D1"

LS_1

LS_14.data <- Read10X(data.dir = "F:/For.Analysis/data/matrix/2405132_Ls2/filtered_feature_bc_matrix")

LS_14 <- CreateSeuratObject(counts = LS_14.data, project = "LS_14", min.cells = 3, min.features = 50)

LS_14@meta.data[,"treatment"]<-"LS"

LS_14@meta.data[,"day"]<-"D14"

LS_14

LS_42.data <- Read10X(data.dir = "F:/For.Analysis/data/matrix/2405132_Ls3/filtered_feature_bc_matrix")

LS_42 <- CreateSeuratObject(counts = LS_42.data, project = "LS_42", min.cells = 3, min.features = 50)

LS_42@meta.data[,"treatment"]<-"LS"

LS_42@meta.data[,"day"]<-"D42"

LS_42

LS_98.data <- Read10X(data.dir = "F:/For.Analysis/data/matrix/2405132_Ls4/filtered_feature_bc_matrix")

LS_98 <- CreateSeuratObject(counts = LS_98.data, project = "LS_98", min.cells = 3, min.features = 50)

LS_98@meta.data[,"treatment"]<-"LS"

LS_98@meta.data[,"day"]<-"D98"

LS_98

Combined <- merge(LS_1, c(LS_14,LS_42,LS_98), add.cell.ids = c("LS_1","LS_14","LS_42","LS_98"),project = "Dev.Chicken")

Combined

saveRDS(Combined, file = "1.Combined.merge.rds")

**# Quality control**

head(Combined@meta.data, 5)

Combined[["percent.mt"]] <- PercentageFeatureSet(Combined, pattern = "^MT-")

VlnPlot(Combined, features = c("nFeature_RNA", "nCount_RNA", "percent.mt"), ncol = 3)

plot1 <- FeatureScatter(Combined, feature1 = "nCount_RNA", feature2 = "percent.mt")

plot2 <- FeatureScatter(Combined, feature1 = "nCount_RNA", feature2 = "nFeature_RNA")

plot1+plot2

Combined.filter <- subset(Combined, subset = nFeature_RNA > 500 & nCount_RNA < 25000 & percent.mt < 15)

Combined.filter

saveRDS(Combined.filter, file = "2.Combined.filtered.rds")

**#Normalization**

Combined.filter <- NormalizeData(Combined.filter, normalization.method = "LogNormalize", scale.factor = 10000)

#Identification of highly variable features (feature selection)

Combined.filter <- FindVariableFeatures(Combined.filter, selection.method = "vst", nfeatures = 2000)

# Identify the 10 most highly variable genes

top10 <- head(VariableFeatures(Combined.filter), 10)

# plot variable features with and without labels

plot1 <- VariableFeaturePlot(Combined.filter)

plot2 <- LabelPoints(plot = plot1, points = top10, repel = TRUE)

plot1 + plot2

**#Scale**

all.genes <- rownames(Combined.filter)

Combined.filter <- ScaleData(Combined.filter, features = all.genes)

**#PCA analysis**

Combined.filter <- RunPCA(Combined.filter, features = VariableFeatures(object = Combined.filter), npcs = 100)

DimPlot(Combined.filter, reduction = "pca")

Combined.filter <- JackStraw(Combined.filter, num.replicate = 100, dims=100)

Combined.filter <- ScoreJackStraw(Combined.filter, dims = 1:100)

JackStrawPlot(Combined.filter, dims = 81:100)

ElbowPlot(Combined.filter, ndims = 100, reduction = "pca") # Select 90

Combined.filter.Harm <- FindNeighbors(Combined.filter.Harm, dims = 1:90) #Reduction to use as input for building the SNN

Combined.filter.Harm <- FindClusters(Combined.filter.Harm, resolution = 0.045) # this function does not need "reduction" ## the smallest cluster does not disapper even when r=0.01; 0.045 works best

Combined.filter.Harm <- RunUMAP(Combined.filter.Harm, dims = 1:90)

**# UMAP**

DimPlot(Combined.filter.Harm, reduction = "umap.harm", label = TRUE, pt.size = 0.05, label.size = 2,raster=FALSE) + NoLegend()+FontSize(x.title = 8, y.title = 8)+theme(axis.text = element_text(size = 8)) + theme(strip.text = element_text(size=8, face="bold"))

ggsave("Combined.filter.Harm.UMAP.combined.tiff", dpi=300, width=2.5, height=2.5)

#reduction = Which dimensionality reduction to use. If not specified, first searches for umap, then tsne, then pca

DimPlot(Combined.filter.Harm, reduction = "umap.harm", split.by="treatment",label = TRUE, pt.size = 0.05, label.size = 2,raster=FALSE) + NoLegend()+FontSize(x.title = 8, y.title = 8)+theme(axis.text = element_text(size = 8)) + theme(strip.text = element_text(size=8, face="bold"))

ggsave("Combined.filter.Harm.UMAP.treatment.tiff", dpi=300, width=3.5, height=3)

DimPlot(Combined.filter.Harm, reduction = "umap.harm", split.by="orig.ident",label = TRUE, pt.size = 0.05, label.size = 2,raster=FALSE) + NoLegend()+FontSize(x.title = 8, y.title = 8)+theme(axis.text = element_text(size = 8)) + theme(strip.text = element_text(size=8, face="bold"))

ggsave("Combined.filter.Harm.UMAP.origident.tiff", dpi=300, width=10, height=3)

saveRDS(Combined.filter.Harm, file = "3.Combined.HarmUmapNotIdentify.rds")

**#Find marker genes**

All.cluster.markers <- FindAllMarkers(Combined.filter.Harm,only.pos = TRUE,min.pct = 0.1)

write.csv(All.cluster.markers,"All.cluster.markers.csv")

**#Cell type annotation**

levels(Combined.filter.Harm)

new.cluster.ids <- c("Myo IIB","SCs","FAPs","Endo","Mac","Myo I","T","Post-Syn","Myo IIA","Pre-Syn","BLCs","Glial")

names(new.cluster.ids) <- levels(Combined.filter.Harm)

Combined.filter.Harm <- RenameIdents(Combined.filter.Harm, new.cluster.ids)

levels(Combined.filter.Harm)

Combined.filter.Harm[["Named.Cluser.id"]] <- Idents(object = Combined.filter.Harm)

DimPlot(Combined.filter.Harm, reduction = "umap.harm", label = TRUE, pt.size = 0.05, label.size = 2,raster=FALSE) + NoLegend()+FontSize(x.title = 8, y.title = 8)+theme(axis.text = element_text(size = 8)) + theme(strip.text = element_text(size=8, face="bold"))

ggsave("Combined.filter.Harm.UMAP.combined.named.tiff", dpi=300, width=3.0, height=2.5)

DimPlot(Combined.filter.Harm, reduction = "umap.harm", split.by="treatment",label = TRUE, pt.size = 0.05, label.size = 2,raster=FALSE) + NoLegend()+FontSize(x.title = 8, y.title = 8)+theme(axis.text = element_text(size = 8)) + theme(strip.text = element_text(size=8, face="bold"))

ggsave("Combined.filter.Harm.UMAP.treatment2.named.tiff", dpi=300, width=4.0, height=2.5)

DimPlot(Combined.filter.Harm, reduction = "umap.harm", split.by="orig.ident",label = TRUE, pt.size = 0.05, label.size = 2,raster=FALSE) + NoLegend()+FontSize(x.title = 8, y.title = 8)+theme(axis.text = element_text(size = 8)) + theme(strip.text = element_text(size=8, face="bold"))

ggsave("Combined.filter.Harm.UMAP.origident2.named.tiff", dpi=300, width=12, height=3)

saveRDS(Combined.filter.Harm, file = "3.1.Combined.HarmUmap.named.Identify.rds")

**#FAP reclustering**

Idents(Combined.filter.Harm) <- "Named.Cluser.id"

FAPs <-subset(Combined.filter.Harm,idents = "FAPs")

FAPs <- FindNeighbors(FAPs, reduction = "harmony",dims = 1:90)

FAPs <- FindClusters(FAPs, resolution = 0.06) #0.06 WORKS BEST

Combined.filter.Harm <- RunUMAP(FAPs, reduction = "harmony", reduction.name="umap.harm", dims = 1:90)

DimPlot(FAPs, reduction = "umap.harm", label = TRUE, pt.size = 0.05, label.size = 2)+ NoLegend()+FontSize(x.title = 8, y.title = 8)+theme(axis.text = element_text(size = 8)) + theme(strip.text = element_text(size=8, face="bold"))+xlim(6,12)+ylim(-10,0)

DimPlot(FAPs, reduction = "umap.harm", label = TRUE, pt.size = 0.05, label.size = 2,split.by = "orig.ident")+ NoLegend()+FontSize(x.title = 8, y.title = 8)+theme(axis.text = element_text(size = 8)) + theme(strip.text = element_text(size=8, face="bold"))+xlim(6,12)+ylim(-10,0)

FAPs.markers <- FindAllMarkers(FAPs,only.pos = TRUE,min.pct = 0.1,recorrect_umi=FALSE) #

write.csv(FAPs.markers,"FAPs.CLUSTERS.markers.csv")

new.cluster.ids <- c("Pre-Adi","Fibro","Multi","Inflam","Adi")

names(new.cluster.ids) <- levels(FAPs)

FAPs <- RenameIdents(FAPs, new.cluster.ids)

levels(FAPs)

FAPs[["sub.cluster.id"]] <- Idents(object = FAPs)

saveRDS(FAPs,"FAPs.1.namedCluster.rds")

**# Compare Adi with Pre-Adi subtype**

Idents(FAPs) <- "sub.cluster.id"

levels(FAPs)

Mature.vs.Adi <- FindMarkers(FAPs,ident.1="Adi",ident.2="Pre-Adi",only.pos = FALSE,min.pct = 0.1,recorrect_umi=FALSE)

write.csv(Aid.vs.Pre-Adi,"DEs. Aid.vs.Pre-Adi.csv")

**# Cell cycle analysis**

cellcycle <-read.csv("F:/Sequencing/CellCycle/CellCycle.genes.x.csv")

s.genes <- cellcycle$S

g2m.genes <- cellcycle$G2

FAPs <- CellCycleScoring(FAPs, s.features = s.genes, g2m.features = g2m.genes, set.ident = TRUE)

FAPs <- RunPCA(FAPs, features = c(s.genes, g2m.genes))

DimPlot(FAPs)+theme(axis.text = element_text(size = 8)) + theme(strip.text = element_text(size=8, face="bold"))+FontSize(x.title = 8, y.title = 8)+theme(axis.text = element_text(size = 8))+theme(legend.text = element_text(size=8))+xlim(6,12)+ylim(-10,0)

ggsave("FAPs.cellcycle.all.tiff", dpi=300, width = 2.5, height = 2)

**#Show marker genes for FAPs subtype**

Idents(FAPs) <- "sub.cluster.id"

features0<- c("COL4A2","CFD","POSTN","COL1A1","KLF5","DPP4","TNC","CCL4","PLIN1","PPARG")

mycolors <- my36colors <- c('#E5D2DD', '#53A85F', '#F1BB72', '#F3B1A0', '#D6E7A3', '#57C3F3', '#476D87','#E95C59', '#E59CC4', '#AB3282', '#23452F', '#BD956A', '#8C549C', '#585658','#9FA3A8', '#E0D4CA', '#5F3D69', '#C5DEBA', '#58A4C3', '#E4C755', '#F7F398','#AA9A59', '#E63863', '#E39A35', '#C1E6F3', '#6778AE', '#91D0BE', '#B53E2B','#712820', '#DCC1DD', '#CCE0F5', '#CCC9E6', '#625D9E', '#68A180', '#3A6963','#968175')

Stacked_VlnPlot(seurat_object = FAPs, features = features0, plot_spacing = 0,group.by = "sub.cluster.id", pt.size = 0.01,vln_linewidth=1,raster=FALSE,colors_use = mycolors)

ggsave("FAPs.SUB.Markers.tiff", dpi=300, width = 3.0, height = 5)

**# Gene ontology analysis (For example)**

library(clusterProfiler)

library(DOSE)

library(ggplot2)

library(enrichplot)

library(org.Hs.eg.db) # Gallus-- Human

library(stringr)

set.seed(1234)

PsDEs <- read.csv("C:/Users/MarkersForFAPs.csv")

geneID <- as.character(PsDEs[,1])

geneID.TOP150 <- geneID[1:101]

GOPsDEs.BP <- enrichGO(geneID.TOP150,

OrgDb = 'org.Hs.eg.db',

ont = "BP",

pAdjustMethod = "fdr",

keyType = 'SYMBOL',

pvalueCutoff = 0.05,

qvalueCutoff = 0.05,

readable = TRUE)

dim(GOPsDEs.BP)

head(GOPsDEs.BP)

GOPsDEs.BP.filter <-simplify(GOPsDEs.BP,cutoff = 0.7,

by = "p.adjust",

select_fun = min)

dim(GOPsDEs.BP.filter)

head(GOPsDEs.BP.filter, 53)

write.csv(GOPsDEs.BP.filter, "C:/Users/FAPs.GO.top100.csv")

a <- c("extracellular matrix organization","cellular response to amino acid stimulus","response to transforming growth factor beta","Rho protein signal transduction","connective tissue development","vascular endothelial growth factor signaling pathway","regulation of chemotaxis","positive regulation of MAP kinase activity","positive regulation of phospholipase activity","cell-cell signaling by wnt")

p1 <- dotplot(GOPsDEs.BP.filter, showCategory = a, font.size=8, x = "GeneRatio")+theme(axis.title = element_text(size=8))+theme(axis.text = element_text(color="black", size=8))+theme(legend.title = element_text(size=8)+theme(legend.text = element_text(size = 8)))+theme(legend.text = element_text(size = 6))+theme(legend.title = element_text(size = 6))+theme(plot.title = element_text(size=8,face = "bold"))+theme(axis.title = element_text(size = 8))+theme(axis.text.x = element_text(angle = 30, size=8))+xlim(NA,0.3)+ggtitle("FAPs")+theme(plot.title = element_text(size=8,face = "bold"))+scale_size_area(max_size = 6)+theme(legend.key.width = unit(0.2,"cm"))+theme(legend.key.height = unit(0.2,"cm"))+scale_y_discrete(labels=function(y) str_wrap(y, width=30))

p1

ggsave('C:/Users/ FAPtop100GO.tiff', dpi=300, width=3.1, height=3.55)

**2. Monocle 3 for pseudotime analysis**

library(monocle3)

library(SeuratWrappers)

set.seed(1234)

LS_98.FAPs_Diet <- DietSeurat(LS_98.FAPs, counts=TRUE,data=TRUE, dimreducs = "umap.harm")

LS_98.FAPs_Diet[["UMAP"]] <- LS_98.FAPs_Diet[["umap.harm"]]

LS_98.FAPs_Diet.cds <- as.cell_data_set(LS_98.FAPs_Diet)

LS_98.FAPs_Diet.cds <- cluster_cells(cd=LS_98.FAPs_Diet.cds, reduction_method = "UMAP")

LS_98.FAPs_Diet.cds <- learn_graph(LS_98.FAPs_Diet.cds,use_partition = TRUE)

LS_98.FAPs_Diet.cds <- order_cells(LS_98.FAPs_Diet.cds, reduction_method = "UMAP")

plot_cells(cds = LS_98.FAPs_Diet.cds, label_cell_groups = FALSE, color_cells_by = "sub.cluster.id", show_trajectory_graph = TRUE, label_branch_points = FALSE, label_leaves = FALSE, label_roots = TRUE, cell_size = 0.5)+theme(axis.title = element_text(size=8))+theme(axis.text = element_text(color='black', size=8))+theme(legend.title = element_text(size=8)+theme(legend.text = element_text(size = 8)))+theme(axis.line.x = element_line(size=0.55))+theme(axis.line.y = element_line(size=0.55))+theme(axis.ticks.x = element_line(size=0.55))+theme(axis.ticks.y = element_line(size = 0.55))+theme(legend.title = element_text(size = 8))+theme(legend.text = element_text(size = 8))+xlim(5,13)+ylim(-9,0)

ggsave("LS_98.FAPs_Diet.PSEDO.tiff", dpi=300, width=3.2, height=2.0)

plot_cells(cds = LS_98.FAPs_Diet.cds, label_cell_groups = FALSE, color_cells_by = "pseudotime", show_trajectory_graph = TRUE, label_branch_points = FALSE, label_leaves = FALSE, label_roots = TRUE, cell_size = 0.5)+theme(axis.title = element_text(size=8))+theme(axis.text = element_text(color='black', size=8))+theme(legend.title = element_text(size=8)+theme(legend.text = element_text(size = 8)))+theme(axis.line.x = element_line(size=0.55))+theme(axis.line.y = element_line(size=0.55))+theme(axis.ticks.x = element_line(size=0.55))+theme(axis.ticks.y = element_line(size = 0.55))+theme(legend.title = element_text(size = 8))+theme(legend.text = element_text(size = 8))+xlim(5,13)+ylim(-9,0)

ggsave("LS_98.FAPs_Diet.PSEDO2.tiff", dpi=300, width=3.2, height=2.0)

LS_98.FAPs_Diet.cds <- estimate_size_factors(LS_98.FAPs_Diet.cds)

LS_98.FAPs_Diet.cds@rowRanges@elementMetadata@listData[['gene_short_name']] <- rownames(LS_98.FAPs_Diet.cds[['SCT']])

RP_genes.module <- c("CFD","COL4A1","COL3A1","DLK1","PDGFRA","COL1A1","FN1")

rowData(LS_98.FAPs_Diet.cds)$gene_name <- rownames(LS_98.FAPs_Diet.cds)

rowData(LS_98.FAPs_Diet.cds)$gene_short_name <- rowData(LS_98.FAPs_Diet.cds)$gene_name

RP_genes.module_cds <- LS_98.FAPs_Diet.cds[rowData(LS_98.FAPs_Diet.cds)$gene_short_name %in% RP_genes.module,label_by_short_name = FALSE]

monocle3::plot_genes_in_pseudotime(RP_genes.module_cds,cell_size = 1.5,color_cells_by="sub.cluster.id")+theme(axis.title = element_text(size=8))+theme(axis.text = element_text(color='black', size=8))+theme(legend.title = element_text(size=8)+theme(legend.text = element_text(size = 8)))+theme(axis.line.x = element_line(size=0.55))+theme(axis.line.y = element_line(size=0.55))+theme(axis.ticks.x = element_line(size=0.55))+theme(axis.ticks.y = element_line(size = 0.55))+ theme(strip.text = element_text(size=8, face="bold.italic"))

ggsave("LS_98.FAPs_Diet.PSEDO2.myogenicGENEs.tiff", dpi=300, width=3.0, height=5)

LS_98.FAPs_Diet.cds <- estimate_size_factors(LS_98.FAPs_Diet.cds)

LS_98.FAPs_Diet.cds@rowRanges@elementMetadata@listData[['gene_short_name']] <- rownames(LS_98.FAPs_Diet.cds[['SCT']])

RP_genes.module <- c("FN1")

rowData(LS_98.FAPs_Diet.cds)$gene_name <- rownames(LS_98.FAPs_Diet.cds)

rowData(LS_98.FAPs_Diet.cds)$gene_short_name <- rowData(LS_98.FAPs_Diet.cds)$gene_name

RP_genes.module_cds <- LS_98.FAPs_Diet.cds[rowData(LS_98.FAPs_Diet.cds)$gene_short_name %in% RP_genes.module,label_by_short_name = FALSE]

monocle3::plot_genes_in_pseudotime(RP_genes.module_cds,cell_size = 1.5,color_cells_by="sub.cluster.id")+theme(axis.title = element_text(size=8))+theme(axis.text = element_text(color='black', size=8))+theme(legend.title = element_text(size=8)+theme(legend.text = element_text(size = 8)))+theme(axis.line.x = element_line(size=0.55))+theme(axis.line.y = element_line(size=0.55))+theme(axis.ticks.x = element_line(size=0.55))+theme(axis.ticks.y = element_line(size = 0.55))+ theme(strip.text = element_text(size=8, face="bold.italic"))

ggsave("LS_98.FAPs_Diet.FN1.PSEDO2.myogenicGENEs.tiff", dpi=300, width=3.2, height=1.8)

**3. RNA velocity analysis using scvelo package in Python**

import rpy2

import anndata

import scvelo as scv

import pandas as pd

import numpy as np

import matplotlib as plt

%load_ext rpy2.ipython

sample_one = anndata.read_loom("C:/Users/Leon2023/OneDrive/Sequencing/Analysis20250101/Loom/loom/2405132_LS4/LS4.loom")

sample_one.var.index.is_unique

sample_one.var_names_make_unique()

sample_one.var_names_make_unique()

sample_one.var.index.is_unique

sample_one

sample_one_index = pd.DataFrame(sample_one.obs.index)

sample_one_index = sample_one_index.rename(columns = {0:'CellID'})

sample_one_index

sample1_obs = pd.read_csv("C:/Users/ LS.FAPs_D98.cellID_obs.csv")

sample_one.obs.index

sample_one = sample_one[np.isin(sample_one.obs.index,sample1_obs["x"])]

sample_one

sample_one.obs.index

sample_one_index = pd.DataFrame(sample_one.obs.index)

sample_one_index = sample_one_index.rename(columns = {0:'CellID'})

sample_one_index

umap = pd.read_csv("C:/Users/ LS.FAPs_D98.cell_embeddings.csv")

cell_clusters = pd.read_csv("C:/Users/LS.FAPs_D98.clusters.csv")

umap_ordered = sample_one_index.merge(umap, on = "CellID")

umap_ordered = umap_ordered.iloc[:,1:]

sample_one.obsm['X_umap'] = umap_ordered.values

umap_ordered.values

sample_one

cell_clusters

cell_clusters_ordered = sample_one_index.merge(cell_clusters, on = "CellID")

cell_clusters_ordered

cell_clusters_ordered = cell_clusters_ordered.iloc[:,1:]

cell_clusters_ordered

sample_one.uns['Cluster_colors'] = cell_clusters_ordered.values

sample_one

sample_one.uns['Cluster_colors']

scv.pp.filter_and_normalize(sample_one)

scv.pp.moments(sample_one)

scv.tl.velocity(sample_one, mode = "stochastic")

scv.tl.velocity_graph(sample_one)

scv.pl.velocity_embedding(sample_one,arrow_size=2, arrow_length=12, xlim=[5,13],ylim=[-9,0], basis='umap', color = sample_one.uns['Cluster_colors'],dpi=600,save="C:/Users/ RNAvelocity.FAP.LSD98.png")

scv.pl.velocity_embedding_stream(sample_one, arrow_size=1.5, basis='umap',color = sample_one.uns['Cluster_colors'],dpi=600, save="C:/Users/ RNAvelocity.STREAM.LSD98.FAP.png",xlim=[5,13],ylim=[-9,0])
